# Simple Patchy-Based Simulators Used to Explore Pondscape Systematic Dynamics

**DOI:** 10.1371/journal.pone.0086888

**Published:** 2014-01-23

**Authors:** Wei-Ta Fang, Jui-Yu Chou, Shiau-Yun Lu

**Affiliations:** 1 Graduate Institute of Environmental Education, National Taiwan Normal University, Taipei, Taiwan, ROC; 2 Department of Biology, National Changhua University of Education, Changhua, Taiwan, ROC; 3 Department of Marine Environment and Engineering, National Sun Yat-sen University, Kaohsiung, Taiwan, ROC; University of Gävle, Sweden

## Abstract

Thousands of farm ponds disappeared on the tableland in Taoyuan County, Taiwan since 1920s. The number of farm ponds that have disappeared is 1,895 (37%), 2,667 ponds remain (52%), and only 537 (11%) new ponds were created within a 757 km^2^ area in Taoyuan, Taiwan between 1926 and 1960. In this study, a geographic information system (GIS) and logistic stepwise regression model were used to detect pond-loss rates and to understand the driving forces behind pondscape changes. The logistic stepwise regression model was used to develop a series of relationships between pondscapes affected by intrinsic driving forces (patch size, perimeter, and patch shape) and external driving forces (distance from the edge of the ponds to the edges of roads, rivers, and canals). The authors concluded that the loss of ponds was caused by pond intrinsic factors, such as pond perimeter; a large perimeter increases the chances of pond loss, but also increases the possibility of creating new ponds. However, a large perimeter is closely associated with circular shapes (lower value of the mean pond-patch fractal dimension [MPFD]), which characterize the majority of newly created ponds. The method used in this study might be helpful to those seeking to protect this unique landscape by enabling the monitoring of patch-loss problems by using simple patchy-based simulators.

## Introduction

Pond integrity is multifaceted with regard to the functions, structures, and variations of ponds based on a management perspective, particularly concerning local and regional biodiversity[1]. Regarding the functional paradigm of farm ponds, Smith et al.[2] examined millions of small water bodies throughout the USA. Compared with large water bodies (e.g., reservoirs and lakes), these small artificial ponds contain many more ecological organisms. In Norway, inland water bodies (e.g., ponds) are also regarded as one aspect of land cover and land use that can serve asa variable in land classification and delineation [3]. Numerous studies have explained the areal increase in species richness by citing an increase in microhabitat heterogeneity. Ponds are vulnerable to disturbances and vanish because of anthropogenic driving forces, which directly influence species distribution. The relative importance of pond configurations and site-specific structures to species is debatable. Several studies have reported that pond patterns (i.e., peripheral anthropogenic land uses) [4]–[6] and pond-size patterns [7], [8] are related to species number and diversity. Pond size is the basic variable used to measure horizontal alterations. Historically, ecologists and geographers have attempted to determine the relationships between spatial size and species richness. As pond size increases, the likelihood of the animals that are new to this particular area will appear increases, thereby increasing species richness. Studies on a similar topic date back to 1921, when an increase in plant species number was observed as the same function of quadrat areas (i.e., the species-area relationship was not just a phenomenon that occurred among plants) [9].

MacArthur and Wilson (1967) proposed an island biogeographic theory to explain spatial variances in avian richness values [10]. They developed the concept of island biogeography, which states that small islands have lower immigration rates and higher extinction rates, thereby resulting in the presence of fewer species compared with those of large islands. Alternatively, large islands, with a larger area and larger populations that are less prone to extinction, support more species. Based on this concept, MacArthur and Wilson suggested that more species inhabit large islands than small islands, and islands adjacent to a mainland contain more species than remote islands do because of higher immigration rates. The concept of island biogeography was also called the area-per-se hypothesis by Connor and McCoy [11]. The area-per-se hypothesis has since been applied to faunal communities, such as breeding birds on islands. This hypothesis was also the major concept used to explain species richness in several studies, which designed water regimes for vernal pools and refuges in earlier decades [12]. Ponds can differ in size for reasons such as drought, drainage, and drawdown of the water surface, which can reduce water regimes over time. Species richness is affected by both natural and anthropogenic influences on pond configuration [13]. The ecological significance of such disturbances might be crucial to aquatic communities. However, the edge effect between aquatic-terrestrial regimes has been completely ignored in species-area studies. An alternative hypothesis that was proposed to explain the area-per-se relationship stated that an increase in habitat heterogeneity accompanies an increase in area. Increased microhabitat diversity allows more species to find an increasing number of niches, resulting in greater species diversity.

Several studies have reported that the areal increase in species richness is caused by an increase in microhabitat heterogeneity. Recent studies have presented new theoretical and empirical methods aimed at creating theories based on transition processes at regional to global scales [14]. Several effects that could be beneficial to ecosystems were observed, such as the(a) area-per-se effect, (b) habitat effect, (c) distance effect, and (d) connectivity effect. The hypotheses require more detailed examinations of ecological theories to investigate anthropogenic influences. If sufficient mudflats and water regimes are maintained, ponds can support more species. Habitat effects can increase the diversity of ponds, and prevent negative anthropogenic influences at the edge of metropolitan areas. For example, if the curvilinear edge of a pond is limited to buildings (built-up areas) and/or highways, the pond is vulnerable to external pressures and damage. In addition, the smaller the distance is between ponds, the more beneficial it is for the dispersal of hydrophilic species.Cluster design should be considered to increase the connectivity of ponds and subsequently prevent the development of pondscapes that are located far from each other, and/or prevent fragmentation that occurs when isolated patches develop in mosaics. Several of these aforementioned hypotheses must be carefully applied in the fields of landscape planning and design.

Forman [15] argued that ecological spatial form is not simply shaped by the effect of area-per-se and isolation patterns. This was only a theoretical hypothesis, which inferred that the pond size used in landscape design affects species richness within a heterogeneous terrain [16]. In addition to area, all variations in ponds can be condensed into four categories that address ecophysical identities: shape, edge, clustering, and connectivity [17], [18]. Given the remarkable diversity and complex compositions of pond configurations, a pondscape is defined in this study as “a series of water surfaces of ponds associated with various surrounding landforms, including farms, creeks, canals, roads, houses, woodlands, and other open spaces,” according to the reports of several researchers[19]–[21].

The field of landscape ecology was comprehensively developed based on the driving forces observed in pondscape studies, using the concepts of shape, edge, clustering, and connectivity. However, landscape ecology studies have seldom detected the anthropogenic influences of the aforementioned spatial driving forces (SDFs), compared with natural science studies [22]–[26]. Predicting the occurrence of anthropogenic factors was crucial in detecting human-induced land-use changes in closed depressions in Brussels [25]. In this study, we developed a series of relationships between pondscapes affected by intrinsic driving forces (patch size and patch shape) and external driving forces (distance from the edge of a pond to the edges of roads, rivers, and canals). This study focused on a unique area of pondscapes containing thousands of individual artificial ponds on the Taoyuan Tableland, which underwent dramatic pond losses during the past century [5], [21], [27]. Becausefew streams exist on the Taoyuan Tableland, the growth of water paddies could not be supported. However, the Taoyuan Tableland has an extremely flat slope (of 1/75–1/100) and the surface soil consists of impermeable red clay;consequently, residents dug ponds to hold water. Cultivation in Taoyuan began in 1680, and there are currently more than 8,000 ponds with a total area of 8,000 ha. At the beginning of the eighteenth century, the residents of Taoyuan constructed a network of ponds and canals for irrigation, which changed the pondscapes. Many ponds were merged into larger ponds and were connected by ditches. In 1964, the Shihmen main canal irrigation system was built to support the southern area of the Taoyuan farmlands through the Shihmen dam, which provided a more stable water supply for agricultural and industrial use. Thus, the surface area and number of ponds were again reduced in the southern region of the Taoyuan Tableland. In addition, because of the population growth and economic development that has occurred in recent years, many of the ponds were filled up and became land used for human activities.

According to a review of studies on the thousands of farm ponds in Taoyuan County, Taiwan, the aforementioned information on pond-structure data has been seldom analyzed. Recently, spatially explicit land-coverdata derived from historical maps were directly linked to biological survey data exhibiting with spatial mapping by incorporating biophysical feedback in models of land-use changes[5], [28]. In this study, the process of these changes and the leading causes of the disappearance of pondscapes were investigated. We first developed an exploratory analysis of pondscape changes during the twentieth century. Two geographic information systems (ESRI ArcGIS10 and ArcView 3.3) and a logistic regression model were then applied to analyze changes based on map-scanning data previously obtained during the period of Japanese colonial rule (1895–1945) and the modern period (the Republic of China's rule [1945 to the present]). Using these methods, we determined the causes of landscape changes that occurred after the Taoyuan main canal irrigation system, which supported the northern region of the Taoyuan farmlands, was constructed during the 1920s. The adoption of a Grand Canal irrigation system might result in an internal restructuring of agricultural land use from traditional farm-pond irrigation to more diversified water sources, such as ditches, canals, and reservoirs. The spatial dependency of pondscape changes associated with northern irrigation systems (Taoyuan main canal) constructed from 1926 to 1960 was identified. Furthermore, the process of urbanization that led to these changes was strategically analyzed using a computer-aided analytical model.

In this study, we (a) examined sensitive metrics, such as configuration metrics, which reduced the number of farm ponds; and (b) analyzed the quantitative limitations of these sensitive metrics. A statistical model was designed to examine how the perimeter effects of farm ponds caused losses on the Taoyuan Tableland. Land-use changes that occurred from 1926 to 1960 were analyzed to demonstrate how enforcing land-use policies might have influenced the direction and magnitude of pondscape changes.

## Materials and Methods

### Description of the Study Area

**Figure 1:**
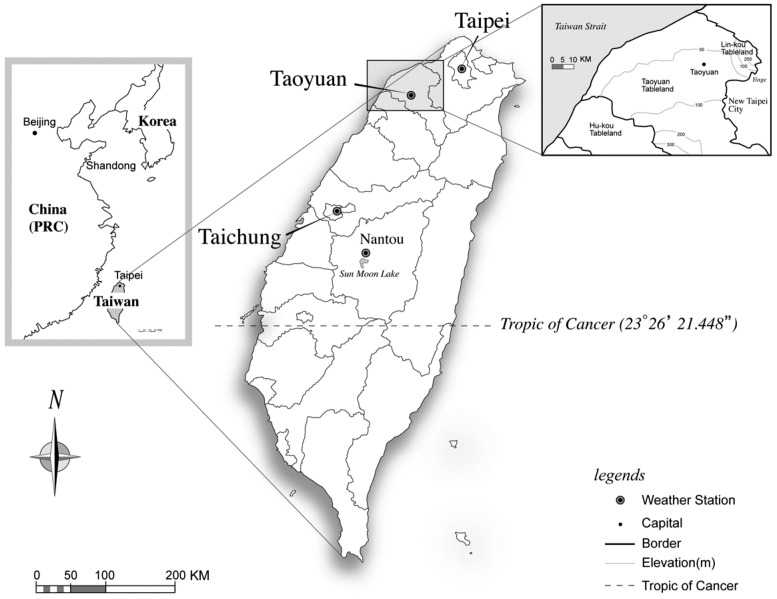
Study area.

The Taoyuan Tableland, located in northwestern Taiwan at approximately 40 km southwest of Taipei, occupies an area of 757 km^2^ based on the elevational contours (Figure 1). “Taoyuan,” which means “peach garden,” is situated in a rich agricultural area where numerous peach orchards existed during the nineteenth century. However, Taoyuan is now a regional market center for commercial and industrial activities and the transportation of goods,and is also considered a new potential aerotropolis. The extent of urban development has increased, and the Taoyuan metropolitan area has become one of the fastest growing areas in Taiwan. Currently, the population of the Taoyuan Tableland is 2,038,000, according to the 2013 census data. This area has a population density of 2,692 persons/km^2^, and the population is increasing at a rate of 2,000–3,000 persons/month. Urbanization influences the urban fabric and also causes changes in pondscape textures. Thus, the constant conversion of farm ponds into urban areas might partially be due to the proximity of Taoyuan County to the highly urbanized Taipei City. To understand the relationships between these phenomena, we set up areas of a matrix to analyze topographic maps by using ArcGIS 10. The total matrix area of the757 km^2^ area covers 11 administrative areas, comprising Shinwu Township, Guanyin Township, Dayuan Township, Jungli City, Yangmei Township, Pingjen City, Luchu Township, Taoyuan City, Bade City, Lungtan Township, and Dashi Township.

### Description of Data Mining

We obtained authorization to scan 60 (4×15 pieces) original paper maps from four periods, using compact recordable disks with a total of 5,000 MB of memory. We digitized paper maps from 1904, 1926, and 1960, which were scanned at the Computer Graphic Center, Department of Geography, Chinese Cultural University (Taipei, Taiwan). The maps we digitized included the Taiwan fort maps from the Japanese colonial period at a scale of 1: 20,000, Taiwan topographic maps (sheet version of the 1926 maps, reprinted in 1998, at a scale of 1: 25,000), and Taiwan geographic maps (sheet version of the 1960 maps, at a scale of 1: 25,000) for three separate years during the twentieth century. Using the TM2 cartographic coordinate system, we established two or more control reference points to calibrate and convert the sheet data to true coordinates by using transformation tools. The digital maps comprised the following types of land use: farm ponds, canals, roads (national highways, provincial highways, and prefectural highways), railways, areas with buildings (built-up areas), and agricultural lands.

### Logistic Regression Model

Landscape change processes were modeled using nonlinear techniques. A logistic regression is a binary classification algorithm determined using a discriminative parametric approach to predict the probability of the occurrence of an event [29], [30]. Logistic regression also involves several predictor variables that are categorical and reflect binary data, such as “success” or “failure.” Logistic regression models are widely used to analyze the driving forces underlying the proximate causes of various events, such as species recolonization [31], [32]and landscape changes [33]–[44]. In this study, the binary value was the “existence” or “disappearance” of farm ponds. We then used the logarithm for calculating the ratio of the probability of “existence” to that of “disappearance” to form a linear regression equation. Because more than one independent variable can influence the probability of “existence” or “disappearance,” the logistic regression model is
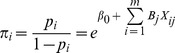
(1)

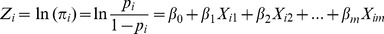
(2)where ln is the natural logarithm, log_e_ (e = 2.71828…), *P i* is the likelihood that pond loss occurs,
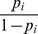
is the “odds ratio,” 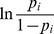
is the log of the odds ratio, or “Logit,” and all of the other components of the model are the same, such as 
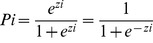
(3)


Whereas 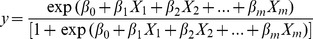 or
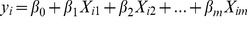
(4)



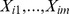
are *m* variables determined by the *i* th pond, and the likelihood is justified by internal driving forces *m*s (i.e., size, shape, etc.). The logistic regression coefficients were interpreted when the variables were correlated to explain the “vanished” conditions. We removed several variables from this analysis because they were only slightly correlated. Several sensitive variables (i.e., those highly correlated with ponds that had disappeared) were selected. Final variable coefficients for which *p*≤.05 were considered significant. In Equation (1), *p_i_* = *P*(*y*_i_ = 1|*x_i1_, x_i2_,…., x_im_*) is a series of independent variables, x*_i1_*, x*_i2_*,…, x*_im_*, which represents the probability of occurrence. The following list gives the assumptions of the logistic regression model: (a) data should be derived from random samples; (b) the dependent variable, y_i_, is assumed to be a function of m independent variables x*_im_* (m = 1, 2, 3,…); (c) the logistic regression model is sensitive to multicollinearity, and the multicollinearity among independent variables influences the standard deviation; (d) the dependent variables used in the logistic regression model are binary variables, and these variables can be presented only as “0” or “1”; and (e) the relationships between independent variables and dependent variables are nonlinear.We randomly added variables to the model, based on the concepts developed by Turner [45], such as (a)nearest neighbor probabilities, (b) the amount of edge between land uses, and (c) the patches of ponds according to size class and land use, and then compared the results with a selected suitable model. IBM SPSS 21.0 software (IBM, Armonk, New York, USA) was used to produce the stepwise regression in this study.

The logistic models were selected using various methods to construct a variety of regression models from the same set of variables: (a) Enter: a process for selecting variables in which all of the variables in a block are entered in only a single step;(b) Forward Selection (Conditional): a stepwise selection method combined with entry testing, based on the significance of the score statistic, and removal testing, based on the probability of a likelihood-ratio statistic and conditional parameter estimates; and (c) Forward Selection (Likelihood Ratio; LR): a stepwise selection method combined with entry testing, based on the significance of the score statistic, and removal testing, based on the probability of a likelihood-ratio statistic and the maximum partial likelihood estimates. Stepwise selection methods were applied in this study to identify covariables in the regression models. This model involves the use of likelihood ratio tests and is applied to prediction situations with a large number of variables to determine which variables are entered and in what order. The difference between Forward Selection (Conditional) and Forward Selection (Likelihood Ratio) is that Forward Selection (Conditional) can be used to perform fast but inaccurate calculations, and Forward Selection (Likelihood Ratio) can be used to perform time-consuming but accurate calculations. The conditional selection might also cause bias, which has been referred to as over fitting in combination with extreme values[46]. One of these methods can be adopted for performing forward stepwise selection to limit the number of covariables and subsequently select coefficients included in the model incrementally. We, therefore, added the final results by using the stepwise selection method based on the Likelihood Ratio approach.

## Results

### “Orgware City” Trends

**Figure 2:**
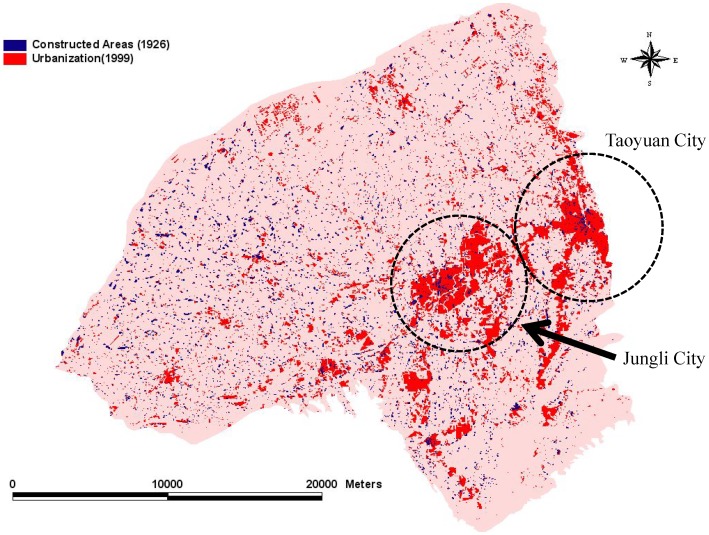
Trends of the progression of built-up areas in 1926.

**Figure 3:**
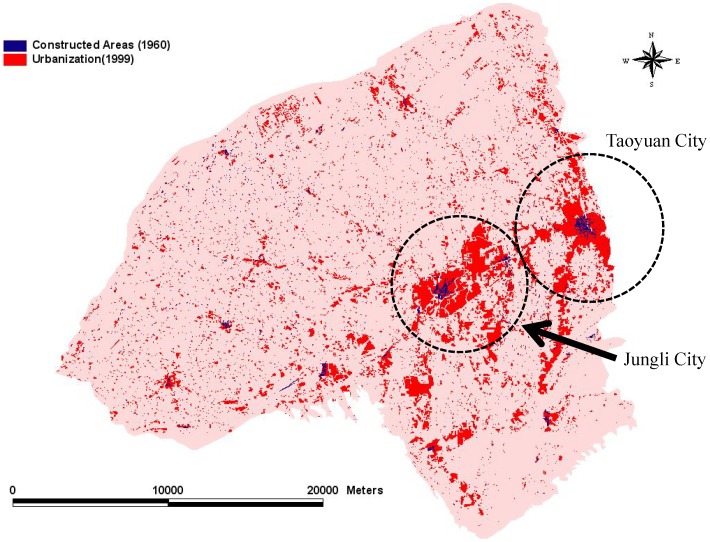
Trends of the progression of built-up areas in 1960.

In this study, we constructed maps according to the topography of the Taoyuan Tableland from 1904 to 1926 and from 1926 to 1960. The location of cities on the Taoyuan Tableland was not obvious in 1904. By contrast, Taoyuan City and Jungli City had begun to form by 1926 (Figure 2), and two “real urban forms” had formed by 1960 because of population concentration (Figure 3). Similar to the scenario outlined in the urbanization model, the population moved from rural villages to cities, such asTaoyuan City and Jungli City, on the Taoyuan Tableland. These two cities are now densely populated areas where concentrated commercial activities occur. Thus, they have become centers of political, economic, and cultural activities. Various stages of concentration, dispersion, invasion, and separation of towns occurred in this area, and cities formed later. In particular, Taoyuan City and Jungli City competed and coexisted with each other, eventually forming an urban corridor along the main transportation lines. These changes followed the “Orgware City” concept and occurred through the interactions of human activities with nature, and nature with transportation (Figures 2 and 3).

### Trends of Farm-Pond Losses

**Figure 4:**
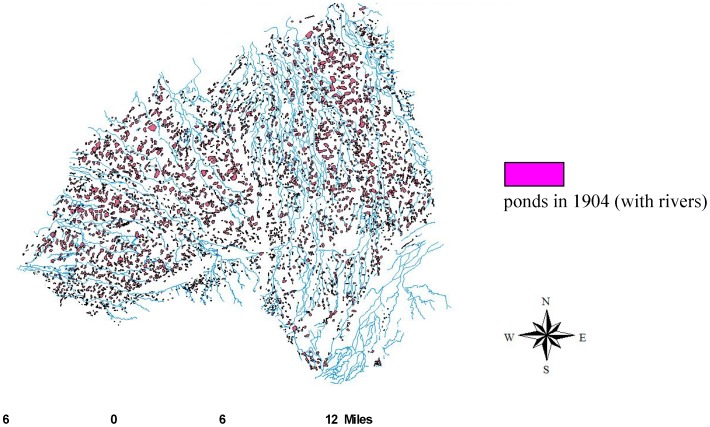
Farm ponds that existed on the Taoyuan Tableland in 1904 (overlapped with local rivers).

**Figure 5:**
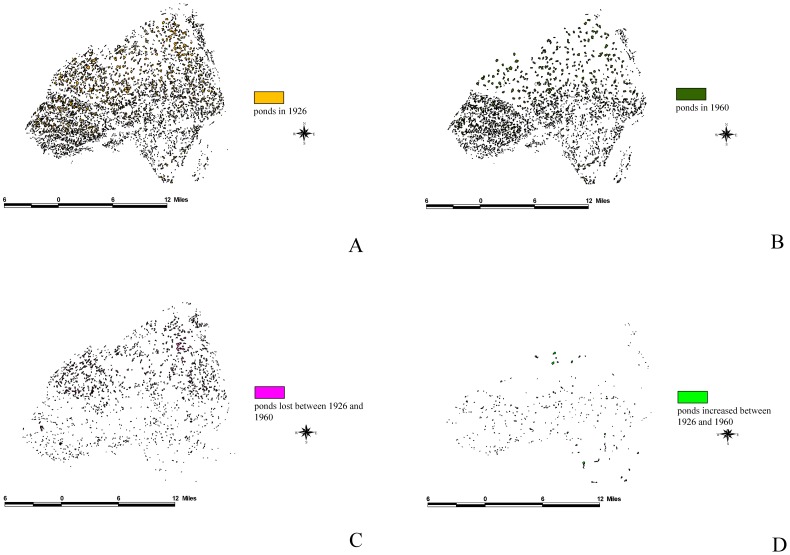
Pondscape changes from 1926 to 1960. (A) Farm ponds that existed on the Taoyuan Table land in 1926. (B) Farm ponds that remained on the Taoyuan Table land in 1960. (C) Trends of losses of farm ponds detected on the Taoyuan Tableland between 1926 and 1960. It is obvious that more farm ponds in the northern region of the Taoyuan Tableland disappeared than in the southern region. (D)Trends of new farm ponds detected on the Taoyuan Tableland between 1926 and 1960.

By comparing Figures 4 and 5A, we discovered that, despite the inconspicuous changes in farm ponds that occurred from 1904 to 1926, there was a decreasing trend in the number of farm ponds. Because the scale of the 1904 maps (1: 20,000) differed from those of other periods (Figure 4), we analyzed only changes from 1926 (1: 25,000; Figure 5A) to 1960 (1: 25,000; Figure 5B). Because the various scales prevented the comparison between 1904 (Figure 4) and 1926 (Figure 5A), we used the map and determined that farm ponds have covered most of the study area since 1904, except for the southeastern corner. In addition, only sporadic villages and residences beyond the forms of cities developed in the early twentieth century.

Figure 5 shows the pattern of the disappearance of farm ponds in northern Taoyuan County, which differed from that of southern Taoyuan County. The number of farm ponds that disappeared was 1,895 (Figure 5C), 2,667 ponds remained, and only 537 new ponds were created (Figure 5D). We observed that the existence of farm ponds closely depended on the use of the Taoyuan main canal irrigation system on the Taoyuan Tableland. However, farm ponds with irregular shapes at the ends of the main canal in the northern region of the tableland disappeared first, whereas the smaller ponds disappeared later.

**Figure 6:**
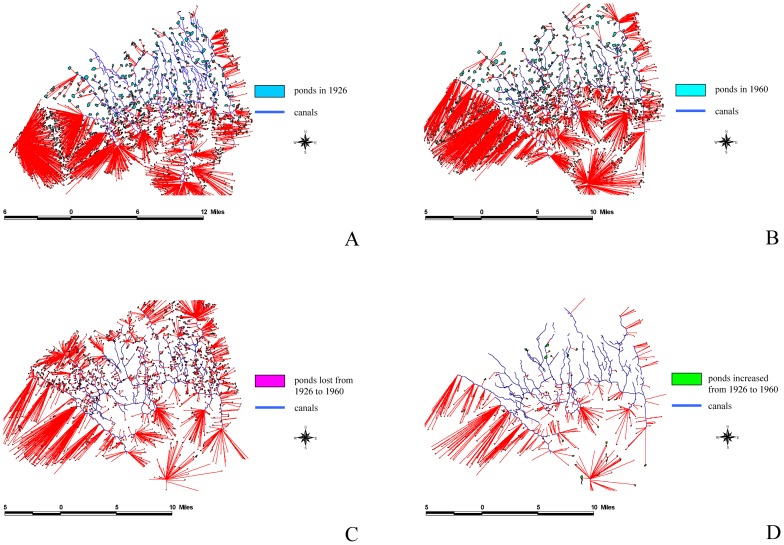
The influence of canals on pondscape changes from 1926 to 1960. (A) Distances between canals and existing ponds in 1926. (B) Distances between canals and remaining ponds in 1960. (C) Distances between canals and lost ponds from 1926 to 1960. (D) Distances between canals and new ponds from 1926 to 1960.

**Figure 7:**
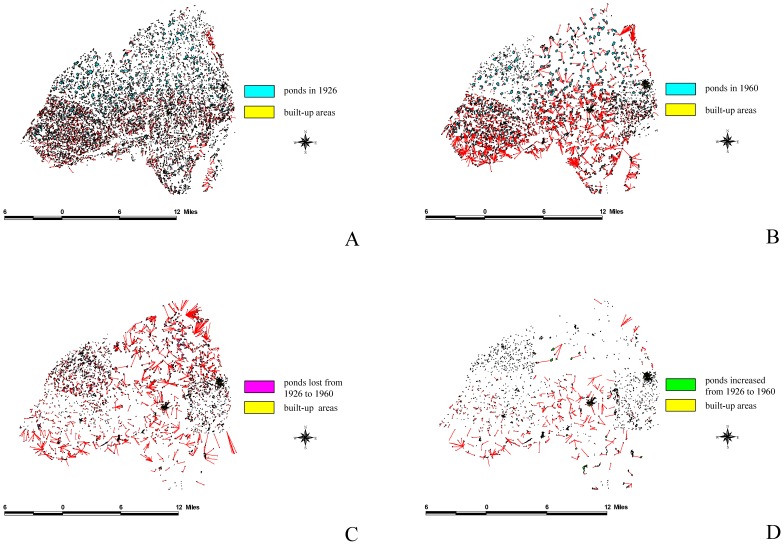
The influence of built-up areas on pondscape changes from 1926 to 1960. (A) Distances between built-up areas and existing ponds in 1926. (B) Distances between built-up areas and remaining ponds in 1960. (C) Distances between built-up areas and lost ponds from 1926 to 1960. (D) Distances between built-up areas and new ponds from 1926 to 1960.

**Figure 8:**
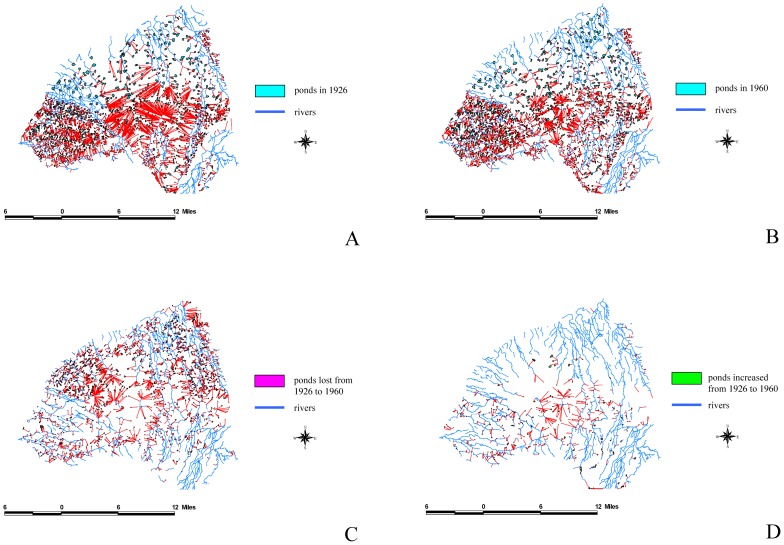
The influence of rivers on pondscape changes from 1926 to 1960. (A) Distances between rivers and existing ponds in 1926. (B) Distances between rivers and remaining ponds in 1960. (C) Distances between rivers and lost ponds from 1926 to 1960. (D) Distances between rivers and new ponds from 1926 to 1960.

**Figure 9:**
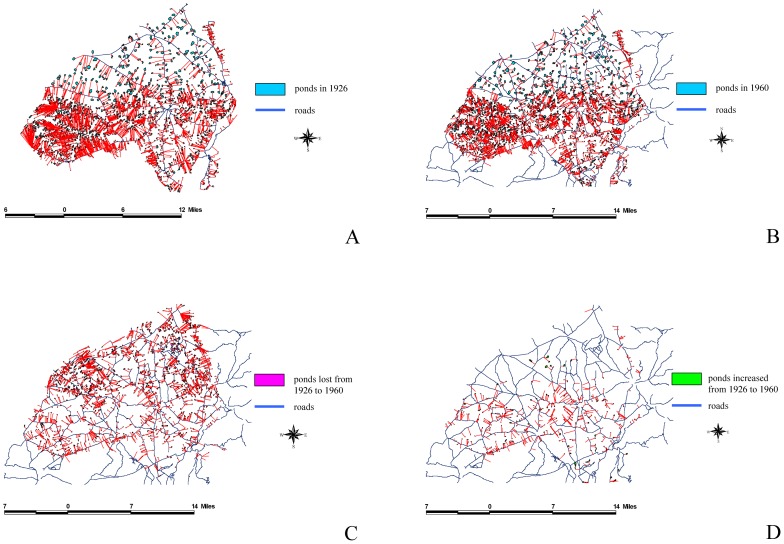
The influence of roads on pondscape changes from 1926 to 1960. (A) Distances between roads and existing ponds in 1926. (B) Distances between roads and remaining ponds in 1960. (C) Distances between roads and lost ponds from 1926 to 1960. (D) Distances between roads and new ponds from 1926 to 1960.

**Figure 10:**
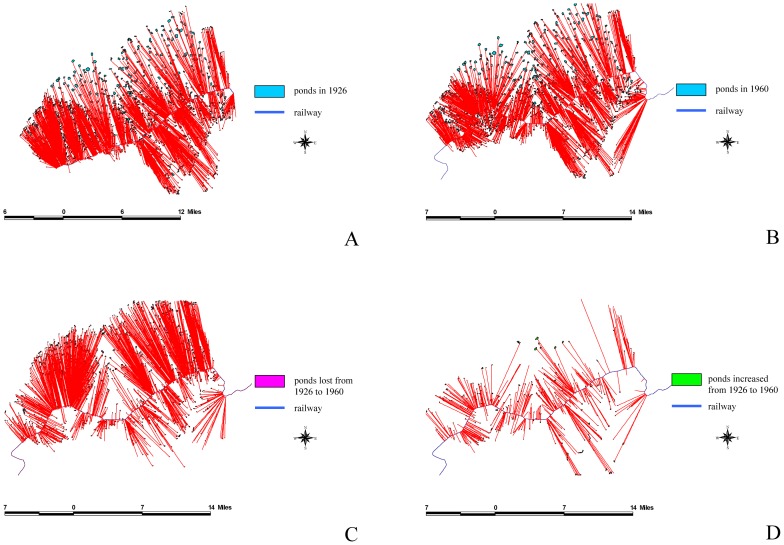
The influence of railways on pondscape changes from 1926 to 1960. (A) Distances between railways and existing ponds in 1926. (B) Distances between railways and remaining ponds in 1960. (C) Distances between railway and lost ponds from 1926 to 1960. (D) Distances between railway and new ponds from 1926 to 1960.

We used ArcGIS to analyze the overlap between the 1926 and 1960 maps to define dynamic patterns and subsequently understand the reasons for these changes. The results of this study indicated that the total area of farm ponds at the peak was 8,800 ha, and accounted for 11.8% of the tableland area. By contrast, the total area of the farm ponds has decreased to 2,898ha because of urbanization, accounting for only 3.8% of the tableland area. Using FRAGSTATS [47], we analyzed the driving forces behind the appearance and disappearance of farm ponds: (a) intrinsic driving forces, which include pond perimeter, pond size, and the mean pond-patch fractal dimension (MPFD); and (b) external driving forces, which included the minimal distance of a farm pond to a canal (Figures 6A, 6B, 6C, and 6D), building (Figures 7A, 7B, 7C, and 7D), river (Figures 8A, 8B, 8C, and 8D), road (Figures 9A, 9B, 9C, and 9D), or railway (Figures 10A, 10B, 10C, and 10D). We then used these relationships to further discuss the driving forces behind changes in farm ponds accompanied bythe respective proximity effects.

The results based on the logistic regression model and using the independent variables indicated the possible reasons for pond changes, which included the area of farm ponds (AREA), the perimeter of farm ponds (PERI), the MPFD, and distances to the nearest artificial structure; specifically,canals (CANAL), buildings (built-up areas; BUILD), roads (ROAD), railways (RAIL), and rivers (RIVER)[45], [48].

### Ponds Lost from 1926 to 1960

The results were divided into 10 groups with approximately 456 cases in each group; see SAV S1 as original datasets. We divided all of the subjects into 10 groups based on their probabilities. Therefore, all of the subjects with a 0.1 probability or lower were placed in the lowest decile, and those with a 0.9 probability or higher were placed in the highest decile. The Hosmer and Lemeshow (HL) test values were determined at Step 2 (23.075, d.f. = 8, *p* = .003>.001) of the Stepwise Forward Selection (Likelihood Ratio) model. The chi-square value (23.075) with a higher *P* value (.003) was accepted for the goodness-of-fit test, compared with that of the other steps (Step1 [224.116, d.f. = 8, *p* = .000], Step 3 [74.601, d.f. = 8, *p* = .000], Step 4 [57.181, d.f. = 8, *p* = .000], and Step 5 [63.340, d.f. = 8, *p* = .000]). By using the HL statistic, we determined that high values indicated large differences between the actual and predicted values for a decile.Therefore, the lower the Hosmer-Lemeshow statistic is, or the higher the corresponding *p*value is, the lower the weakness of fit of the logistic regression model is.

At Step 2, we labeled the standardized regression coefficients as “Beta” (B). The B of PERI (*p* = .000) was equal to 0.002, and the constant was equal to 0.262 (*p* = .000). In this model, a unit of change in PERI led to a 0.002-fold change in the occurrence rate with significance. However, neither MPFD, AREA, CANAL, BUILD, RIVER, ROAD, nor RAILproduced a significant change in the respective occurrence rates. According to the data listed in Table 1, we present the logistic regression model as

**Table 1:** Binomial logit model of pond losses for the period 1926∼1960. Unit of observation: individual ponds in 1960.

		B	S.E.	Wals	df	Significance	Exp(B)	Exp(B) at a 95.0% confidence level	
								Lower level	Upper level
Step 1^a^	RAIL	.000	.000	284.656	1	.000	1.000	1.000	1.000
	Constant	1.102	.055	398.575	1	.000	3.011		
Step 2^b^	PERI	.002	.000	304.498	1	.000	1.002	1.002	1.003
	RAIL	.000	.000	374.463	1	.000	1.000	1.000	1.000
	Constant	.262	.072	13.316	1	.000	1.299		
Step 3^c^	PERI	.003	.000	373.709	1	.000	1.003	1.003	1.003
	CANAL	.000	.000	202.802	1	.000	1.000	1.000	1.000
	RAIL	.000	.000	325.626	1	.000	1.000	1.000	1.000
	Constant	−.519	.090	33.250	1	.000	.595		
Step 4^d^	PERI	.003	.000	369.346	1	.000	1.003	1.003	1.003
	CANAL	.000	.000	256.434	1	.000	1.000	1.000	1.000
	RIVER	.000	.000	69.760	1	.000	1.000	1.000	1.000
	RAIL	.000	.000	262.406	1	.000	1.000	1.000	1.000
	Constant	−.972	.105	85.276	1	.000	.378		
Step 5^e^	PERI	.003	.000	368.965	1	.000	1.003	1.003	1.003
	CANAL	.000	.000	247.843	1	.000	1.000	1.000	1.000
	RIVER	.000	.000	66.615	1	.000	1.000	1.000	1.000
	ROAD	.000	.000	15.229	1	.000	1.000	1.000	1.000
	RAIL	.000	.000	274.600	1	.000	1.000	1.000	1.000
	Constant	−1.059	.108	96.551	1	.000	.347		

Note 1:

a. Selected into a variable: railroad in Step 1.

b. Selected into a variable: PERI in Step 2.

c. Selected into a variable: CANALin Step 3.

d. Selected into a variable: RIVER in Step 4.

e. Selected into a variable: ROAD in Step 5.

Note 2:

1) AREA: the area of pond; PERI:the perimeter of pond; MPFD: Mean Pond Fractal Dimension;CANAL: distances of farm ponds from nearest canals;BUILD: distances of farm ponds from nearest buildings (built-up areas);RIVER: distances of farm ponds from nearest rivers;ROAD: distances of farm ponds from nearest roads;RAIL: distances of farm ponds from nearest railways.

2) The equation to Mean Pond Fractal Dimension (MPFD):

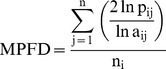
(5) a_*ij*_ =  the area of pond *ij* (in m2).

*n_i_* = the number of the pond *ij.*

*p_ij_*  =  the perimeter of pond *ij* (in m).

Level: CLASS, LANDSCAPE

Units: None

Range:1<MPFD<2

Description: MPFD reflects shape complexity across a range of pond size. It equals 2 times the logarithm of pond perimeter (m) divided by the logarithm of pond area (m^2^) (Li &Reynolds, 1994). MPFD approaches 1 for shapes with very simple perimeters such as circles or squares, and approaches 2 for shapes with highly convoluted and plane-filling perimeters.

Logit (π) = 0.262 + 0.002PERI

Based on this model, we concluded that the larger the perimeter of a pond was, the more easily it was lost, even if the cause was not obvious. By contrast, we determined that the shape of ponds significantly influenced the loss rate.

### Ponds Created from 1926 to 1960

The results were divided into 10 groups with approximately 310 cases in each group; see SAV S2 as original datasets. We divided all of the subjects into 10 groups based on their probabilities. The HL value was determined at Step 3 (12.420, d.f. = 8, *p* = .133>.001) of the Stepwise Forward Selection (Likelihood Ratio) model. The chi-square value (12.420) with a higher *p*value was accepted for the goodness-of-fit test, compared with that of the other steps (Step 1 [56.017, d.f. = 8, *p* = .000] and Step 2 [16.291, d.f. = 8, *p* = .000]). As stated previously, by using the HL statistic, we determined that high values indicated large differences between the actual and predicted values for a decile. Therefore, the lower the HL statistic is, or the higher the corresponding *P* value is, the lower the weakness of fit of the logistic regression model is.

At Step 3, we labeled the standardized regression coefficients as B. The B of PERI (*p* = .000) was equal to 0.002, the B of MPFD was equal to −14.130 (*p* = .000), the B of RIVER was equal to 0.000 (*p* = .006), and the constant was equal to 20.261 (*p* = .000). Therefore, a unit change in PERI led to a 0.002-fold change in the occurrence rate, the MPFD led to a-14.130-fold change in the occurrence rate, And RIVER led to a 0-fold change in the occurrence rate. Neither AREA, CANAL, BUILD, ROAD, nor RAIL changed the occurrence rate. From Table 2, we present the logistic regression model as

**Table 2:** Binomial logit model of pond increases for the period 1926∼1960. Unit of observation: individual ponds in 1960.

		B	S.E.	Wals	df	Significance	Exp(B)	Exp(B) at a 95.0% confidence level	
								Lower level	Upper level
Step 1^a^	MPFD	−17.735	1.206	216.370	1	.000	.000	.000	.000
	Constant	25.574	1.628	246.803	1	.000	127866882315.499		
Step 2^b^	PERI	.002	.000	24.436	1	.000	1.002	1.001	1.002
	MPFD	−13.807	1.424	94.077	1	.000	.000	.000	.000
	Constant	19.716	1.978	99.364	1	.000	365137138.357		
Step 3^c^	PERI	.002	.000	25.378	1	.000	1.002	1.001	1.002
	MPFD	−14.130	1.436	96.871	1	.000	.000	.000	.000
	RIVER	.000	.000	7.648	1	.006	1.000	1.000	1.000
	Constant	20.261	1.999	102.743	1	.000	629546300.185		

Note 1:

a. Selected into a variable: MPFD in Step 1.

b. Selected into a variable: PERI in Step 2.

c. Selected into a variable: RIVER in Step 3.

Note 2: Variables are defined in the footnotes to Table 1.

Logit (π) = 20.261 + 0.002PERI – 14.130 MPFD

Based on this model, we concluded that the larger the perimeter of a pond was, the more likely the pond would be affected by human activities, even if the cause was not obvious. Consistent with the previous model, we determined that the shape of ponds significantly influenced the occurrence rate.

## Discussion

### Pond Loss as an Intrinsic Function

The number of farm ponds that disappeared was 1,895 (37%), 2,667 ponds remained (52%), and only 537 (11%) new ponds were created within a 757 km^2^ area in Taoyuan, Taiwan between 1926 and 1960. According to the studies conducted by Heath and Whitehead[49], similar cases of pond loss were observed in the United Kingdom (U.K.). As an island nation, 55% of the ponds in the U.K.present in 1870 had disappeared by 1960, with the greatest loss occurring between 1920 and 1960. In addition, Western Europe also demonstrated a loss rate of 55% between 1900 and 1990 [50]. Systematic evidence has been presented for pond loss caused by infilling, which occurred mainly during land development periods in the twentieth century[51]. This type of land-form change, together with the extension of urban land uses and the extension of transportation land uses,negatively affected pond loss [52].

In this study, we examined large packages of mapped landscape metrics in pond loss by using multivariate statistical approaches to investigate phenomena that involved spatial interactions and neighborhood characteristics [52]–[54]. We determined proximate causes by using only intrinsic metrics, such as PERI and the MPFD, in addition to external driving forces (i.e., distance between the pond edge and road edge) based on historical images of pond losses. However,we determined that the main reasons for the loss of ponds or for the increase in the number of ponds were pond intrinsic factors, such as pond perimeter and pond shape, rather than external driving-force factors, such as the distance to roads, rivers, and canal edges. According to the first model, Logit (π) = 0.262 + 0.002PERI, an increase in pond perimeter led to the loss of ponds; however, according to the second model, Logit (π) = 20.261 + 0.002PERI – 14.130 MPFD, an increase in pond perimeter also led to an increase in the number of ponds.

In this study, pond losses and pond increases were considered to be independent phenomena that should be discussed separately. Generally, larger pond perimeters indicated increased likelihood of pond loss between 1926 and 1960; in addition, larger pond perimeters indicated increased likelihood that new ponds would be created between 1926 and 1960. However, a large perimeter was closely associated with circular shapes (low value of MPFD) beyond irregular and curvilinear shapes (high value of MPFD), which characterize the majority of ponds created during these periods.

### Beyond External Driving Forces

This result of intrinsic functions beyond external driving forces was determined beyond our hypotheses, as claimed by several authors [34], [35], [39]. Studies conducted in an agricultural area of northern France have reported that small, man-made ponds were more often affected and disappeared more rapidly compared with larger ponds[52].Using the forward logistic regression approach, they also determined that pond persistence was consistently and positively affected by marsh, grasslands, and dune shrubs, and negatively affected by arable and urbanized lands. Braimoh and Onishi [39] claimed that rising demand for urban land and the construction of new roads changed the rural land located at the periphery of existing built-up areas[55]. This might have caused substantial losses of critical natural resources and increased landscape fragmentation. They discovered that one of the determinants (i.e., the proximity to roads) encouraged landform changes to original settlements and residential areas [34], [35], [55].

In comparing the results of this study to those of other related or similar studies, considering pond loss and the increase in the number of pondsin Taiwan as a land-use function driven by external driving-force factors, such as the distance to roads, rivers, canal edges, etc., is difficult to verify. Although the proposed model was developed using limited GIS maps from 1926 to 1960, it appeared to more suitably describe the function driven by intrinsic forces than that driven by landform changes based on the forward logistic regression. The results indicated that one of the major driving forces was pond shape. The final result also indicated that pond shape and pond perimeter were the major reasons that an increasing trend in the number of ponds from 1926 to 1960 was observed.

We also discovered that a curvilinear shape parameter was associated with several pond losses, in contrast with the increase in the number of ponds with a round shape. The more regular the surface of a pond patch was, the less likely the pond was lost. Therefore, curvilinear pond losses were more likely to occur during early agricultural periods because they were replaced by a canal irrigation system.This represents a crucial dynamic of irrigation types observed within the region.

By contrast, we discovered that SDFs, such as the distance of farm ponds from canals (CANAL), buildings (built-up areas; BUILD), rivers (RIVER), roads (ROAD), and railways (RAIL), were not major variables. The neighborhood characteristics of canals, buildings (built-up areas), rivers, roads, and railways presented in this paper were, therefore, not suitable for explaining pond-loss patterns on a regional scale. However, although land use from surrounding pondscapes became diverse and heterogeneous, the configuration of the lost ponds might not be statistically significant. Therefore, further ecological field research is necessary to interpret these pondscape dynamics more comprehensively. Finally, assumptions were made to validate the normality and linearity in this study for simulating land-use and land-cover changes [56], [57].

### Morphology Determined Landscape

Because of the characteristics of the mildly sloping gradients of the Taoyuan Tableland, morphology has long been at work in this region. In attempting to study the effects of retrospective influences on the increase in the number of ponds, we also discuss the morphologic influences determined by the landscape. For example, the results of this study suggested that the change in the condition of a pond, which can be either lost or increased, is related to the inherent morphology of the pond, such as size complexity and perimeter. These parameters are related;for example, if we consider two shapes with similar sizes, and one shape is characterized by a regular perimeter and the other is characterized by a fractal perimeter by a high MPFD value, the irregular perimeter is expected to be longer than that of the regular perimeter. Therefore, if the MPFD was greater than 1, the pond was highly formed as a curvilinear shape. Thus, we determined the conditions of the types of ponds that increased in number, which were characterized bya more circular and less curvilinear shape, and a lower value of MPFD. The reason that a farmer dug out a more circular pond than an irregularly shaped pond might have been that a circular pond was designed to avoid soil erosion from the pond fringe. In addition, the influences of an irregular pond fringe from potential nonpoint source pollution on nearby farm ponds should be considered. Because the original function of farm ponds was agricultural, farmers were more likely to secure water quality, quantity, and safety than focus on other functional uses between 1926 and 1960. Regarding these anthropogenic activities on the tableland, the results of this study indicated that the MPFD decreased as the number of ponds increased.

### Land Policies on Pond Conservation

Numerous factors affect farm-pond conversion. When the benefits off arm ponds do not exceed the benefits brought by changes in advanced land use, farm-pond conversion occurs. In addition to the aforementioned spatial parameters, we also considered the effect of land policy. Because the policies on farm land transformation were determined by private landowners, the farm-pond policies lifted the restrictions on farmland trades, thereby facilitating a more direct spatial transition of ponds. In this area, most farmland trades are associated with ponds located adjacent to urban-fringe lands. The reasons that the topic of farm-pond conversion should be emphasized are related to urbanization. Because the great demand for constructed land use in this situation decreases the number of ponds, the government should urge farmers to create more new ponds characterized by large, circular shapes because of the benefits associated with these types of ponds, according to the findings of this study. This could protect the integrity of the agricultural environment and subsequently secure the irrigation function, as well as provide efficiency and equity from a balanced perspective of natural resources.

### Conclusion

Pondsare defined as an artificial construction made to impound water through the use of a dam or an embankment, or by excavating a pit or a hole. This type of wetscape provides a unique scenic view of a landscape located between nature and humanity that has existed for over a hundred years in the Taoyuan Tableland of northwestern Taiwan. However, these wetscapes are being lost because of public construction, anthropogenic activities, and land use for economic development.

The relationship between pond loss and pond shape was a robust predictor for changes in landforms. We developed a series of models for pondscapes affected by intrinsic driving forces (patch size and patch shape) and external driving forces (distance from the edge of a pond to the edges of roads, rivers, and canals). The final results reflected the effect of various intensive driving forces on pond changes during specific periods in the history of the study area, and we concluded that these changes occurred because of pond intrinsic factors, such as pond size, and that pond-loss rates were affected by pond shape rather than by external driving forces. The models, based on a probabilistic method for calculations using binary logistic regression, were used to determine the effects of landform changes, which are represented by proximate causes underlying SDFs. In assessing the configurations of pondscape areas, however, assessing the loss rates and patterns of and identifying the effects of SDFs on land-use changes are crucial because they might indicate that the scientific rigor of generic theories of conceptual models must be reduced before these models can be used to explain land-use changes [14], [58]. Several SDFs, however, are too simplified to explain their proximate causes and consequences, and are difficult to use to support theories. In addition, a simple extrapolation of the trends possesses less predictive power because of the predictive uncertainties in space and/or time[59]. Therefore, the reasons for land-use changes should be discussed in both simple and general contexts[60]. This might be facilitated using logistic stepwise regression as a simple patchy-based approach to explore pondscape dynamics for monitoring pond-loss problems, once people begin to emphasize the importance of protecting this unique pondscape on the Taoyuan Tableland.

## Supporting Information

Sav S1 **The SPSS file indicated as pond disappeared (0) and pond remained (1) associated with parameters to support this study. (SPSS SAV, original datasets).**
(SAV)

Sav S2 **The SPSS file indicated as pond increased (0) and pond remained (1) associated with parameters to support this study. (SPSS SAV, original datasets).**
(SAV)
